# Cerebrospinal fluid neopterin: an informative biomarker of central nervous system immune activation in HIV-1 infection

**DOI:** 10.1186/1742-6405-7-15

**Published:** 2010-06-03

**Authors:** Lars Hagberg, Paola Cinque, Magnus Gisslen, Bruce J Brew, Serena Spudich, Arabella Bestetti, Richard W Price, Dietmar Fuchs

**Affiliations:** 1 Department of Infectious Diseases, Sahlgrenska University Hospital, University of Gothenburg; SE 41685 Sweden; 2 Department of Infectious Diseases, San Raffaele Scientific Institute, Milan, Italy; 3 Departments of Neurology and HIV Medicine, St. Vincent's Centre for Applied Medical Research St. Vincent's Hospital, University of New South Wales, Sydney, Australia; 4 Department of Neurology, University of California San Francisco, San Francisco, CA, USA; 5 Division of Biological Chemistry, Biocenter, Innsbruck Medical University, Innsbruck, Austria

## Abstract

HIV-1 invades the central nervous system (CNS) in the context of acute infection, persists thereafter in the absence of treatment, and leads to chronic intrathecal immunoactivation that can be measured by the macrophage activation marker, neopterin, in cerebrospinal fluid (CSF). In this review we describe our experience with CSF neopterin measurements in 382 untreated HIV-infected patients across the spectrum of immunosuppression and HIV-related neurological diseases, in 73 untreated AIDS patients with opportunistic CNS infections, and in 233 treated patients.

In untreated patients, CSF neopterin concentrations are almost always elevated and increase progressively as immunosuppression worsens and blood CD4 cell counts fall. However, patients with HIV dementia exhibit particularly high CSF neopterin concentrations, above those of patients without neurological disease, though patients with CNS opportunistic infections, including CMV encephalitis and cryptococcal meningitis, also exhibit high levels of CSF neopterin. Combination antiretroviral therapy, with its potent effect on CNS HIV infection and CSF HIV RNA, mitigates both intrathecal immunoactivation and lowers CSF neopterin. However, despite suppression of plasma and CSF HIV RNA to below the detection limits of clinical assays (<50 copies HIV RNA/mL), CSF neopterin often remains mildly elevated, indicating persistent low-level intrathecal immune activation and raising the important questions of whether this elevation is driven by continued CNS infection and whether it causes continued indolent CNS injury.

Although nonspecific, CSF neopterin can serve as a useful biomarker in the diagnosis of HIV dementia in the setting of confounding conditions, in monitoring the CNS inflammatory effects of antiretroviral treatment, and give valuable information to the cause of ongoing brain injury.

## Introduction

### History

The AIDS dementia complex (ADC) or HIV-associated dementia (HAD) was recognized as a novel central nervous system (CNS) disorder early in the AIDS epidemic [1] and subsequently linked to a pathological substrate of HIV encephalitis (HIVE) [2]. Not long after this recognition, a number of investigators sought objective laboratory biomarkers in the cerebrospinal fluid (CSF) that might provide insight into pathogenesis and also aid in diagnosis and disease staging, which were otherwise based on the constellation of clinical signs and symptoms and their impact on functional capacity. This search paralleled similar efforts to find blood markers of systemic disease that could more clearly predict systemic disease progression and prognosis. Indeed, the CSF studies examined some of the same biomarkers that were being studied in blood as systemic disease markers. One of these was the pteridine metabolite, neopterin, the blood and urine concentrations of which were found to predict systemic disease progression [3]. Neopterin was noted to be elevated in the CSF of HIV-infected patients, and particularly high levels were reported in patients with ADC/HIVE, suggesting that this might be a useful CNS disease marker [4-6]. The origin of neopterin in activated macrophages also fit with emerging recognition of the central role of these cells in ADC/HIVE pathogenesis [7,8].

However, interest in neopterin and other soluble immunological biomarkers in blood waned with the development and widespread clinical use of quantitative assays of HIV-1 RNA that provided a valuable practical guide to the pace of disease progression and the effects of treatment. In parallel attention to CSF immunological biomarkers, including neopterin, declined after it was shown that HIV RNA levels could be measured in the CSF of most untreated patients and that high levels could often be detected in ADC/HIVE [6,9,10]. Attention also shifted to other quantitative methods, including anatomical and functional neuroimaging and neuropsychological testing, to advance diagnosis and patient characterization [11].

More recently, several factors have converged to suggest that it might be worthwhile to revisit immunological CSF biomarkers in general, and neopterin, in particular. One of these again parallels considerations of systemic HIV disease and relates to the renewed appreciation of the importance of immune activation in systemic disease pathogenesis and progression [12]. A number of studies show that immunological markers on blood T cells can provide prognostic information beyond that of the blood viral load and CD4+ T cell count; in fact, at least one more recent study shows that blood neopterin can also add to prognosis even when these other markers are taken into account [13]. Another is the difficulty in diagnosis of ADC/HIVE in many patients currently presenting with neurological symptoms and signs within a background context of drug use, psychiatric disorders, homelessness and socioeconomical deprivation which, unfortunately, also frequently reduce access and capacity to adhere to combination antiretroviral therapy (cART), leaving these patients with pre-existing neurological disease particularly vulnerable to progressive HIV disease, including ADC. These patients may elude diagnosis as illustrated in one of the case examples described below. Additionally, if there is a rationale for tailoring drug combinations for more effective CNS treatment, it may be important to predict and diagnose ADC/HIVE by more objective means than ordinary clinical examination which can miss diagnosis or by neuropsychological testing which may be affected by other conditions. Finally, with successful viral suppression by antiviral treatment, there remains the important question of whether neurological injury still continues as a result of persistent CNS infection and immune activation, explaining the high prevalence of neurocognitive impairment in treated patients [14]. CSF neopterin might provide a convenient and reliable measure of ongoing brain pathology. Thus, CSF neopterin measurement may contribute to addressing these several issues.

### Approach of this Review

In this review we examine changes in CSF neopterin concentrations in the different stages of systemic HIV infection and HIV-related neurological disease in untreated patients and the impact of treatment. To examine and illustrate these issues, we have aggregated a cross-sectional experience derived from four clinical sites (Gothenburg, Sweden; Milan, Italy; San Francisco, California USA; and Sydney, Australia) that span a broad range of subjects who have been examined in the context of natural history, treatment and clinical studies. Some of these patients have been reported as part of smaller previous reports [6,15-19], but they are now collected together and supplemented by unpublished experience in order to provide a broader picture of CSF neopterin changes in HIV infection.

We will first briefly review the biology of neopterin and its use as an indicator of macrophage activation in HIV CNS infection and disease. We will then describe our experience with this measurement in the aggregate cross-sectional cohort and also present some longitudinal subject examples before considering what these findings indicate and how neopterin might be used in the future. While CSF neopterin has been known to be elevated in HIV infection and further increased in ADC/HIVE and CNS opportunistic infections for more than two decades [4,5], most studies characterizing the concentrations of this pteridine have focused on small groups or compared only restricted subject groups.

## Biology of CSF Neopterin

**Figure 1:**
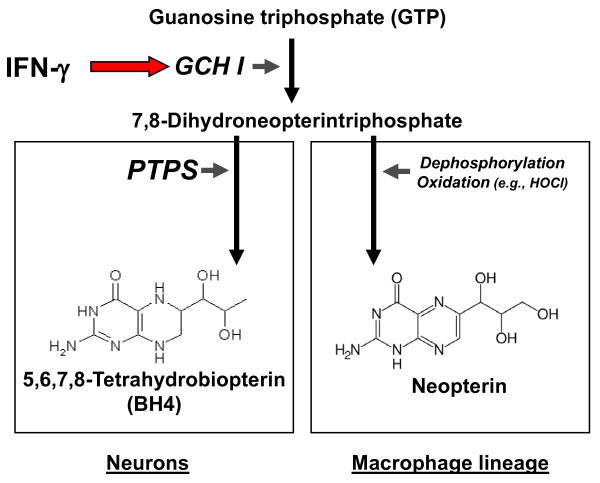
**Induction of neopterin formation in brain cells**. Pro-inflammatory cytokines like interferon-γ (IFN-γ) induce expression of GTP-cyclohydrolase I in various brain cells. As an intermediate product 7,8-dihydroneopterin-triphosphate is produced which is further converted by pyruvoyl-tetrahydropterin synthase (PTPS) to form 5,6,7,8-tetrahydrobiopterin (BH4), the cofactor of several aromatic amino acid monooxygenases that are involved in the production of tyrosine, L-DOPA, serotonin and nitric oxide. Different from neurons, monocytic cells possess only low constitutive activity of PTPS. Thus, 7,8-dihydroneopterin-triphosphate does not undergo conversion to BH4, rather it is dephosphorylated and oxidized to neopterin in non-enzymatic reactions.

Neopterin is a biochemical product of the guanosine triphosphate pathway that is both cell-restricted and inducible by immune-inflammatory stimuli. It is produced primarily in monocyte/macrophage and related cells and the most important stimuli are interferons, especially Th1-type cytokine interferon-γ (IFN-γ) (Figure 1) [3]. Other cells and cytokines have only limited potential to induce neopterin formation *in vitro*, but importantly tumor necrosis factor-α (TNF-α) can accelerate neopterin synthesis when initiated by IFN-γ [20]. By contrast, immunosupressants such as cyclosporin-A, and Th2-type cytokines including interleukin-4 and -10 counteract the production of neopterin [21]. The same is true for anti-inflammatory compounds including certain HMG-CoA reductase inhibitors (statins) and salicylic acid [22]. The cytokine-induced formation of neopterin appears to be part of the antimicrobial and antineoplastic action of macrophages [23].

A strong correlation also exists between neopterin levels and the release of reactive oxygen species (ROS) by macrophages [24,25], which might be of particular relevance in neurodegeneration. Neopterin also induces the expression of pro-inflammatory signal transduction element nuclear factor-κB (NF-κB) [26,27], and the expression of cytokines and inflammatory mediators [28], and intercellular adhesion molecule-1 (ICAM-1) [29]. Production of relevant amounts of neopterin is species-restricted and occurs only in the monocytes/macrophages and astrocytes of primates but not in other animal species. In these cells neopterin is biosynthesised at the expense of 5,6,7,8-tetrahydrobiopterin (BH4), the necessary cofactor of amino acid monoxygenases [30,31]. BH4 is also cofactor of the cytokine-inducible enzyme nitric oxide synthase (iNOS), one of the most important cytotoxic reactions of macrophages stimulated by IFN-γ. However, in human monocytic cells expressing iNOS the concentrations of BH4 are diminished and thus iNOS activity may lead to the accumulation of highly toxic and vasoconstrictory peroxynitrite at the expense of vasodilatory nitric oxide. Moreover, neopterin likely participates in several other important molecular biological pathways involving macrophages and oxidative stress.

There is often a good correlation between blood and CSF neopterin concentrations in patients with HIV infection. An early study also demonstrated a significant correlation between blood neopterin concentrations and the loss of brain tissue expressed as the ventricle-brain ratio measured by computed tomography [32]. The parallel production of systemic and CNS neopterin and ROS production may contribute to brain tissue injury in this setting. At the same time, other cytotoxic compounds may accumulate as a result of CNS immune activation, when tryptophan is degraded via the kynurenine pathway. Interferon-γ, some other cytokines as well as the HIV regulatory protein tat and nef induce activity of the enzyme indoleamine 2, 3-dioxygenase simultaneously with neopterin release [33,34]. This enzyme degrades the essential amino acid tryptophan to N-formyl-kynurenine, which in macrophages is further converted to the neurotoxic substance, quinolinic acid [35]. Thus, neopterin production appears to be part of the cascade of neurotoxic processes in HIV infection, and hence can also serve as a biomarker of these processes.

A practical further aspect of relevance in considering the use of CSF neopterin as a disease biomarker in comparisons to others, including the cytokines that regulate its production, is its stability in biological fluids, due to its rather polar chemical character, ready diffusibility, and long half-life. By contrast many cytokines (including, for example, IFN-γ) have short half-lives with biological activities that rely on effects on neighbouring cells in close proximity rather than at a distance and that might not be as well reflected in lumbar CSF. Neopterin appears to provide a more stable indicator of the aggregate macrophage activation in the CNS compartment. It is also easily and reliably measured with commercially available ELISA or RIA (both from BRAHMS, Hennigsdorf, Germany) that have been shown to yield comparable results [36]. Additionally, these assays have a large dynamic range that encompasses the concentrations encountered in physiological and pathological states in human and other primates. Like all such CSF markers, lumbar CSF concentrations cannot distinguish a regional source within the brain or indeed how much was produced within the brain and how much in the leptomeninges. The lumbar CSF reflects the aggregate intrathecal activity after diffusion and intermixing.

## CSF Neopterin Across the Spectrum of HIV Infection

To provide a view of the CSF neopterin changes across the spectrum of HIV infection and HIV-related CNS injury within the context of other biomarkers, we examined a cross-sectional sample derived from four clinical centers that included HIV seronegative subjects, untreated neuroasymptomatic HIV-infected subjects grouped according to blood CD4+ T cell, ADC neurological diagnoses, two groups of treated HIV-patients and five groups with CNS opportunistic diseases.

The 53 HIV-seronegative subjects in San Francisco who volunteered for study lumbar puncture (LP) as controls were derived from a similar background to the HIV-infected subjects in San Francisco (mean age 43.9 years); 43 (81%) were male, similar to the proportion in the HIV-infected subjects. The untreated HIV-infected subjects without overt neurological disease (referred to as *neuroasymptomatics*, NA) were stratified by blood CD4+ T cell counts and included: 53 subjects with CD4+ counts <50 cells/μL (mean age 38.9); 69 subjects with CD4+ counts 50-199 cells/μL (mean age 38.6); 69 with counts 200-349 cells/μL (mean age 38.8); and 108 with CD4+ counts >350 cells/μL (mean age 37.5). Untreated patients with ADC were divided into 30 with Stage 1 (mean age 38.9) and 53 with Stage 2-4 severity (mean age 40.1). Treated subjects included 150 with plasma HIV RNA suppressed below 50 copies/mL (referred to as treatment *successes*) (mean age 43.4) and 83 with >50 copies/mL (treatment *failures*) (mean age 45.3) after >6 months of treatment. The 73 subjects with CNS opportunistic diseases (referred to henceforth as opportunistic infections, OIs) included 16 with progressive multifocal leukoencephalopathy (PML), 13 with cytomegalovirus encephalitis (CMV-E), 18 with toxoplasmic encephalitis (toxo), 16 with cryptococcal meningitis (crypto) and 10 with primary CNS lymphoma (PCNSL). CSF neopterin was measured by either EIA or RIA using the BRAHMS kit and following the manufacturer's instructions. The assays were performed in Innsbruck (Gothenburg, Milan, Sydney and some of San Francisco samples) and San Francisco (majority of San Francisco samples including all HIV negative samples); while formal quality control comparison between those two laboratories was not done, samples in this and subsequent studies performed in duplicate at both sites were in close agreement (<12 percent variance). Multiple group comparisons were analyzed by Kruskal-Wallis test and Dunn's multiple comparison post hoc tests, two-group comparisons used the Mann Whitney test, while correlations among variables across groups used Spearman's test, all performed with Prism 5 (GraphPad Software).

**Figure 2:**
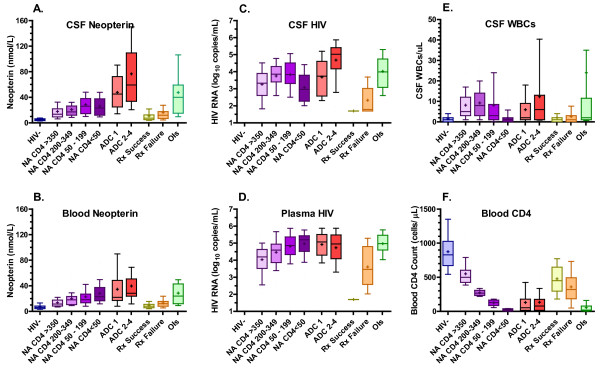
**Cross-sectional analysis of CSF neopterin in HIV disease in the context of other CSF and blood measurements**. Included are 53 HIV-seronegative volunteers; untreated HIV positive neurologically asymptomatic (NA) subjects; 53 with CD4+ counts <50 cells/μL (mean age 38.9); 69 subjects with 50-199 cells/μL (mean age 38.6); 69 with counts 200-349 cells/μL (mean age 38.8); and 108 with CD4+ counts >350 cells/μL. Untreated patients with ADC were divided into 30 with Stage 1, and 53 with Stage 2-4. Treated subjects included 150 with plasma HIV RNA suppressed below 50 copies/mL (treatment *successes*) and 83 with >50 copies/mL (treatment *failures*) after >6 months of treatment. The OI group included 73 patients with CNS opportunistic diseases (see text). The boxes show the 25-50^th ^quartile with median bar and mean +, while the whiskers show the 10-90^th ^quartile. A. CSF neopterin. Overall ANOVA P < 0.0001, Dunn's post hoc comparisons showed that HIV- group differed from all HIV+ groups (P < 0.001 except Sucesses P < 0.5); ADC 2-4 differed from all NAs (P < 0.01- 0.001) but not from ADC 1 group; the ADC 1 group differed from the NA CD4 >350 (P < 0.05) and 200 - 349 (P < 0.001) but not from other NA groups. The treated successes differed both from all the untreated HIV-infected groups (P < 0.001) and the HIV negatives (P < 0.05), while the treated failures also differed from the untreated HIV-infected (P < 0.05- 0.001), except those with CD4 >350, and from the HIV- (P < 0.001). The OI group differed from the NAs with CD4>200 and treated groups but not from those with lower counts or from ADC groups. B. Plasma neopterin. Statistical analysis was similar to CSF except that ADC 2-4 differed only from the two higher CD4 NAs (P < 0.01- 0.001) and the ADC 1 only from the CD4 >350, and the treatment successes did not differ from the HIV seronegatives while the failures did (P < 0.001). C. CSF HIV RNA. D. Plasma HIV RNA. E CSF WBC counts. F. Blood CD4+ T cell counts. Abbreviations: HIV-, HIV seronegative control group; NA, neurologically asymptomatic; ADC, AIDS dementia complex; Rx Success, treated with plasma suppression to <50 copies HIV RNA per mL; Rx Failure, treated with continued plasma viremia with ≥ 50 copies HIV RNA per mL.

The results of the CSF neopterin determinations on this aggregate of 741 subjects are shown in Figure 2 along with blood neopterin, CSF and plasma HIV RNA levels and CSF white blood cell (WBC) counts to provide context.

### CSF Neopterin in Systemic Disease Progression

CSF neopterin was elevated compared to HIV- controls (mean 5.3, SD 2.2 nmol/L) in untreated HIV infection across the spectrum of CD4+ T cell decline. Indeed, CSF neopterin increased as blood CD4+ cells fell, rising from a mean of 17.9 nmol/L (SD 17.7) in those with CD4+ counts >350 cells/μL, to 21.0 nmol/L (SD 14.2) with CD4+ cell counts of 200 - 349 cells/μL, and seeming to plateau in those with 50 - 199 and < 50 cells/μL, with means of 28.7 and 26.2 (SDs 17.2 and 17.1), respectively (Figure 2A). Clearly, HIV infection led to almost universal intrathecal immunoactivation as measured by neopterin, and this increased as immunosuppression worsens and CD4+ T cells fell to below 200 cells/μL. In part these increases in CSF neopterin paralleled those of CSF HIV RNA levels, and indeed across all of the untreated HIV-positive subjects without opportunistic disease the CSF neopterin correlated with the CSF HIV RNA levels (p < 0.0001, Spearman r = 0.4742). However, a notable deviation in this parallel rise was found in the group with CD4+ T cells below 50 cells/μL in whom the HIV RNA levels fell below the neuroasymptomatic groups with higher CD4+ T cells (Figure 2C), while the neopterin did not. These changes in CSF neopterin were not simply a reflection of a general increase in CSF inflammation, though this may provide a partial explanation, since the CSF WBC counts actually decreased to nearly normal levels in subjects with <50 CD4+ cells/μL (Figure 2E) while CSF neopterin remained elevated.

The changes in blood neopterin (Figure 2B) showed a similar increase with falling CD4+ T cells, and overall correlated with the CSF neopterin (p < 0.0001, Spearman r = 0.567), though the levels were lower in the blood, particularly in the ADC groups. This increase in blood neopterin also paralleled the plasma HIV RNA levels (p < 0.0001, r = 0.433) (Figure 2D)

### CSF Neopterin in ADC

This group included patients defined by impairment in their cognitive-motor functional status in daily life and confirmed by bedside examination (rather than test performance on formal neuropsychological testing) and classified as ADC stages 1-4 as previously defined [37]. In brief this staging rates patient's functional disturbance from *mild *but definite impairment in daily activities (Stage 1), to *moderate *impairment with inability to perform the more demanding aspects of daily life (Stage 2), *severe *with major intellectual or motor incapacity and slowing (Stage 3), or *end stage *disease with nearly vegetative state and only rudimentary comprehension and responses (Stage 4)

In the patients with ADC stage 1-4, there was a notable jump in CSF neopterin (Figure 2A) compared to the neuroasymptomatic groups, including those with CD4 counts below 200 with whom they might most appropriately be compared (Figure 2F). This was seen in patients with Stage 1 and particularly Stage ≥2 ADC who exhibited a marked increase in this CSF marker (means of 47.8 and 76.6 nmol/L, with SD of 27.5 and 55.1 nmol/L, respectively) compared to the non-ADC groups, while the two ADC groups did not differ from each other. The stage 2-4 ADC patients had higher CSF HIV RNA levels than the other groups (Figure 2C). The ADC groups had higher CSF WBC counts than the non-ADC group with <50 CD4+ cells, but other HIV+ groups had similar WBC counts without such neopterin increase, which clearly indicates that CSF neopterin in ADC was not caused by the CSF pleocytosis. Indeed, comparison of CSF neopterin and CSF WBC counts across the HIV+ groups shows a clear dissociation and indicates that the changes in CSF neopterin with ADC were not simply part of a non-specific inflammatory response.

Blood neopterin was also higher in the ADC patients, but did not show the same increase as CSF. Thus, whereas the increases in blood and CSF levels of neopterin were of similar magnitude in the neuroasymptomatics (for example, mean CSF and blood levels of patients with <50 CD4+ cells/μL were 26.2 and 28.2 nmol/L, respectively), the increase in blood neopterin in the ADC patients was notably less marked than in the CSF. Blood neopterin was higher in the ADC 2-4 than in the NA with CD4+ cell counts ≥ 200, but not in those below 200.

### CSF neopterin in CNS OIs

**Figure 3:**
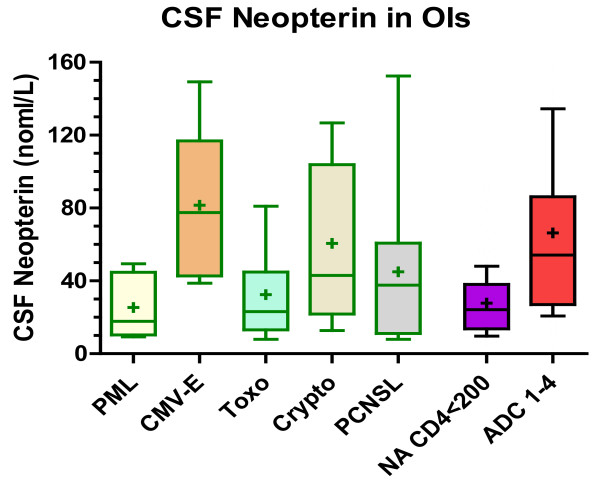
**CSF neopterin concentrations in the 73 subjects with CNS opportunistic diseases (OIs) included 16 with progressive multifocal leukoencephalopathy (PML), 13 with cytomegalovirus encephalitis (CMV-E), 18 with toxoplasmic encephalitis (toxo), 16 with cryptococcal meningitis (crypto) and 10 with primary CNS lymphoma (PCNSL), and for comparison, neuroasymptomatic HIV positive (NA) subjects with <200 blood CD4 counts (collapsed from two groups in Figure 2) and ADC 1-4 (also collapsed from two groups in Figure 2)**. The box and whiskers and statistical methods are as described for Figure 2. The CSF neopterin in the PML group differed from the CMV-E (P < 0.001) and ADC 1-4 group (P < 0.01) but not from the other OI groups or from the NA group. The CMV encephalitis patients had the highest levels and in addition to differing from the PML group, differed from the toxoplasmosis patients (P < 0.01) and neuroasymptomatics (P < 0.001), but not the ADC group. The toxoplasmosis group, in addition to differing from the CMV group also differed from the ADC group (P < 0.05) but not the other OIs or NAs. The cryptococcal meningitis group differed from the NA with low CD4 T cells/μl (P < 0.05) while the PCNSL group did not differ from the other groups.

Because data on the CSF concentrations in CNS OIs in HIV infection are limited, we included subjects with five different OIs in this analysis. None of the patients were on antiretroviral treatment at the time of CSF collection. The diagnosis was confirmed by positive CSF PCR for JC virus in progressive multifocal leukoencephalopathy (PML) and for cytomegalovirus (CMV) in CMV encephalitis, by response to treatment for toxoplasmosis, by CSF cryptococcal antigen or culture, and for primary CNS lymphoma (PCNSL) by histological confirmation or presumptively by combining CSF Epstein-Barr virus PCR, thallium 201 SPECT and lack of response to antitoxoplasmic treatment (Figure 3). As a group, the CSF neopterin in the OIs differed from: the HIV negatives (P < 0.001); both treatment groups (P < 0.001); NAs with blood CD4 counts >350 cells/μl (P < 0.001); and NAs with CD4 200-349 (P < 0.05); But they did not differ from the NAs with CD4 50-199 or <50 or from either ADC group.

Because of the heterogeneity of this OI aggregate group, we further examined the individual OIs and compared them to two groups with similar CD4 counts, the NAs with <200 and the ADC 1-4 (both of these groups derived from combination of two groups from the initial analysis). As shown in Figure 3, two of the OIs, PML and toxoplasmosis had relatively low CSF neopterin levels, and indeed these did not differ from the 152 subjects in the NA group. By contrast the CSF neopterin was highest overall in the CMV-E group and intermediate in the cryptococcal and PCNSL groups, both with broad range of values, with only the toxoplasmosis group differing from the ADC group (P < 0.05).

Thus, overall, OIs can confound diagnosis of ADC using CSF neopterin, particularly in the case of CMV encephalitis which may also be difficult to distinguish by neuroimaging and non-focal clinical findings, emphasizing the importance of CSF CMV PCR in this differential diagnosis. The other OIs can usually be distinguished by clinical and neuroimaging findings. The relatively low CSF neopterin in PML is consonant with the paucity of inflammation pathologically. Since none of these patients exhibited clinical or radiographic immune reconstitution inflammatory syndrome (IRIS), it will be of interest in the future to examine whether neopterin or other CSF immune inflammatory markers might help to understand and clinically distinguish and monitor this disorder [38].

### CSF Neopterin in Treated Patients

Treated patients were defined as those receiving at least three antiretroviral drugs (cART). They were divided into success and failures, according to plasma HIV-1 levels above or below 50 copies/mL. CSF neopterin was, in general, markedly reduced in the two treated patient groups compared to the untreated subjects, showing that combination therapy has a potent effect on intrathecal immunoactivation. However, as previously reported [18,19], these reductions fell short of reaching the levels of the HIV seronegative controls. Thus, the successfully treated group had a mean CSF neopterin concentration of 10.8 nmol/L (+/-10.3 SD) and the failure group 16.2 nmol/L (+/-18.5) compared to the HIV- control concentration mean of 5.3 (+/-2.2). This indicates a state of continued intrathecal immunoactivation in these treated patients, and with considerable variability. Whether this continued activity relates to persistent CNS HIV infection despite CSF HIV RNA levels below the standard level of laboratory detection of 50 copies/mL or to a persistence of immune activation due to some other cause is an important topic of study. We have shown elsewhere that these low levels of CSF neopterin may relate to continued replication that can be demonstrated with more sensitive viral detection methods [39].

**Figure 4:**
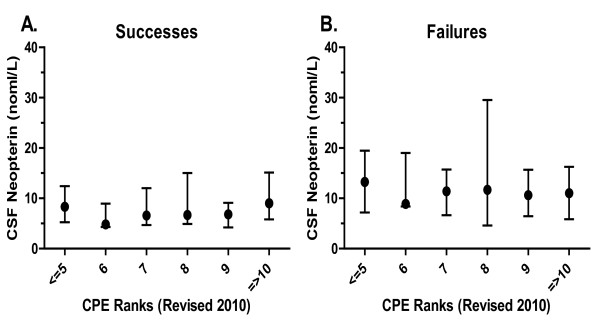
**CSF neopterin in A. Successes and B. Failures**. Relation to the revised 2010 CPE rank scores [40]. There were no significant differences in CSF among these groups, nor was there a correlation when all ranks were considered as a continuous variable. Symbols show the medians and lines the intraquartile range for each group. Additionally, no correlation was found using the older CPE score system (not shown) [41].

While the ANOVA analysis that include all of the groups did not show a difference between the successes and failures, a simple comparison between these two groups suggested a significant difference (p = 0.0004 using Mann Whitney test) consistent with the view that more effective viral control had an effect on CSF neopterin. However in neither group did the levels of CSF neopterin clearly relate to the penetration and efficacy of their antiviral drugs, at least as measured by the CNS penetration-effectiveness (CPE) score or rank as proposed and recently revised by Letendre and colleagues [40,41]. We analyzed the possible effects of the aggregate CNS penetration and efficacy of the patients' antiviral drugs on CSF neopterin for both the success and failure groups, and found no correlation either across the entire group (Spearman's test) or between CPE rank groups. Figure 4 shows the analysis using the modified 2010 CPE rank score, and the earlier CPE score gave similar results.

These results also bring up the issue of normal levels of CSF neopterin. For this study our controls were taken from a population with a similar range of risks and background conditions as the HIV-infected subjects. This may account for the higher mean level in this group than in other control group studies. In an earlier study of 24 healthy volunteers CSF, neopterin concentrations ranged between 3.2 and 5.5 nmol/l (mean 4.2 nmol/L) with the RIA method (Henning/BRAHMS Berlin) [42]. In 47 control individuals regarded as healthy (aged 18-76 years), for whom CSF analysis was done because of headache or vertigo but infection and other diseases were excluded as much as possible (CSF albumin and cell count were normal), the mean neopterin concentration was 4.0 nmol/L (+ 2 SD = 5.9 nmol/L, again using the RIA method)[43]. The normal levels of CSF neopterin increase with age [42], though we found no significant age effect among either the HIV negative controls or the infected neuroasymptomatic groups, both of which contained a relatively restricted age distribution (Spearman's test, not shown). A later Swedish patient cohort with similar inclusion criteria included 55 individuals and found the mean neopterin concentration to be 4.6 nmol/l (SD, 0.7 nmol/L with the same RIA method). Pooling these three studies provided a population of 126 individuals with a mean CSF neopterin value of 4.2 nmol/l (SD, 0.8 nmol/l), and likely approximates the normal concentrations; using this value + 2SD, this would provide a clinical upper limit for normal CSF neopterin of 5.8 nmol/L encompassing 97.5% of values. The 53 HIV- subjects shown in Figure 2 were recruited as control volunteers for comparison with HIV-infected patients; some were substance abusers, and hence inclusion was not as stringent with the objective of approximating the HIV+ population rather than the ideal 'norm'. In this group the mean neopterin concentration was higher than the Swedish controls at 5.3 nmol/L and the variance was greater (SD, 2.2 nmol/L) (by EIA method, Henning Berlin). These measurements were also all performed in San Fancicsco, and it is possible that this biased the results. This higher, less stringent figure was used for comparison in this analysis of HIV effects. However, whichever of these control values one uses, the HIV+ subjects, including those treated effectively, all had higher CSF neopterin concentrations (P < 0.01 - 0.001).

## Longitudinal Case Examples of Treatment

**Figure 5:**
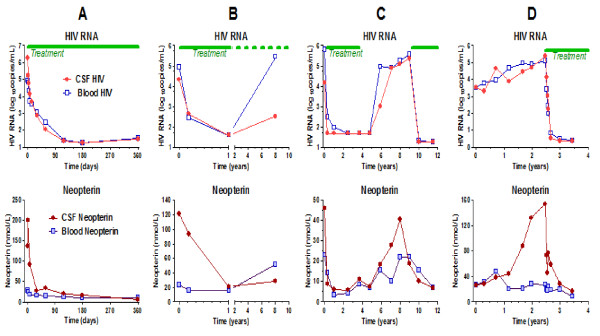
**Four subjects studied longitudinally**. For each of the four subjects (A-D) the top panel shows the HIV RNA concentrations and treatment intervals and the bottom panel the CSF and blood neopterin levels. The symbol definitions in the two A panels apply to all four subjects.

Four longitudinal case examples shown in Figure 5 further illustrate the CSF neopterin response to treatment and emphasize some of the dynamics of its change with disease evolution and treatment. In the figure each case (A - D) shows the changes in CSF and blood HIV RNA levels in the upper panel and the CSF and blood neopterin in the lower panel.

The first patient (A) illustrates the rapid decrease in CSF immune activation and HIV RNA in some patients with ADC treated with potent cART. It also shows the dissociation of intrathecal and systemic macrophage activation at baseline. The response to therapy shows that the high level of CSF neopterin was 'driven' by HIV infection, since it improved as quickly as viral replication was inhibited.

**Patient A **was 33 years old when he presented with Stage 2 ADC in January, 2000 with both cognitive and motor (including spastic gait) impairment. This was his presenting manifestation of HIV infection which was diagnosed at the same time with a blood CD4+ T cell count of 133 cells per μL. He was treated with abacavir, 3TC, nevirapine, and ritonavir-boosted indinavir with rapid HIV RNA response in both blood and CSF (top panel). After a transient increase, his high CSF neopterin also fell rapidly, and over the year of follow-up reached a near normal level (6.4 nmol/L). While his blood neopterin was also elevated and fell, the magnitude at baseline and subsequent change were far less than the CSF. Over the same period he improved clinically and was able to return to acting school, albeit with mild residual gait stiffness; his performance measured by an aggregate Z score n four quantitative neurological performance tests (QNPZ-4) improved from -4.56 to -1.86 [44].

The second patient (B) again illustrates the potent effects of cART on CSF neopterin. It also illustrates a phenomenon reported in other 'failing' patients - CSF HIV RNA levels may remain disproportionately reduced in the face of drug resistance and poor adherence [45]. Also, as in the larger failures group in Figure 2, the CSF neopterin was also reduced in this setting, though remaining above that of HIV seronegatives.

**Patient B **was 47 years when diagnosed with ADC Stage 2 in January, 2000, again with a substantially higher CSF than blood neopterin level. Dementia was his presenting manifestation of HIV infection and the blood CD4+ T cell count was 130 cells per μL The CSF neopterin response was a little slower then in the previous case when he was treated with ritonavir-boosted indinavir, zidovudine and lamivudine. It remained elevated at 21.3 nmol/L at one year when the CSF (and blood) HIV RNA had reached the limit of detection (40 copies per mL). Subsequently, his treatment adherence varied (dashed line in top panel) and his plasma HIV RNA rose above his pre-treatment level. The CSF HIV RNA level, though detectable, did not rise proportionately, nor did his CSF neopterin which remained modestly elevated, but not to the baseline level. Over the effective treatment period his mental status improved clinically.

The third patient (C) again illustrates the dissociation of CSF and blood neopterin levels, even without overt CNS disease, and the response to cART over a very long period and after treatment interruption.

**Patient C **was 51 year in January, 1997 when diagnosed with a symptomatic primary HIV infection with a CD4 cell count of 250 cells per μL. Treatment was given immediately with indinavir, zidovudine and lamivudine. He has been followed for 12 years with yearly lumbar punctures, including a period of treatment, drug holiday, and resumed treatment. As he stopped his treatment, the CSF neopterin rose to 40.5 nmol/L and when new treatment with efavirenz, abacavir and lamivudine was given, the CSF neopterin concentration fell to just above normal (6.9 nmol/L).

The final patient (D) illustrates a steady rise in CSF neopterin that was dissociated from his relatively stable blood neopterin, proportionally exceeded his log_10 _CSF HIV RNA increase and preceded his clinical presentation. This suggests not only an increase in CNS infection, but a switch in its character to a type that associates with brain injury. In this case, the change in CSF neopterin might have served as a helpful indicator of the development of ADC.

**Patient D **was 41 years old when he first entered a longitudinal natural history (the sentinel neurological cohort, SNC) study in July, 2002. He had a history of drug abuse and psychiatric disease, both of which obscured his underlying HIV-related neurological impairment as he began to develop neurological disease over the second year of follow-up; this also contributed to his refusal to begin cART. While his blood and CSF HIV RNA levels gradually increased over the initial two years of his course, this also did not lead to starting therapy. His CSF neopterin rose steeply during the second year of follow-up at a time when was judged neurologically stable until he presented with increasing confusion and was diagnosed with ADC Stage 1 and a blood CD4+ T cell count of 267 cells per μL (his nadir). He was hospitalized and began treatment with zidovudine, 3TC and nevirapine. His CSF neopterin decreased rapidly, then more gradually, reaching 6.4 nmol/L at the end of follow up. CSF and plasma RNA were at the limit of detection. He also recovered clinically with eventual restoration to normal activities with his QNPZ-4 improving from -2.28 before treatment to 0.45 after.

## Pathobiological Implications of CSF Neopterin Changes in HIV

Together the presented data, along with earlier studies, show that neopterin is produced in the intrathecal space (higher CSF than blood concentrations) and that increased CSF concentrations of this pteridine indicate a nearly universal state of enhanced macrophage activation within the CNS in HIV infection. Its elevation with infection and rapid decrease with treatment show that it is ultimately driven by HIV infection. However, in ADC patients, the levels of CSF neopterin rise above those in neuroasymptomatic patients with comparable systemic and CSF HIV RNA concentrations and pleocytosis. One speculation is that to some extent neopterin is produced in the meningeal and perivascular spaces in relation to local infection, but that in ADC and its underlying substrate, HIVE, the character of infection and its capacity to produce neopterin changes as infected and uninfected macrophages and microglia are activated. Extending this hypothetical framework, the augmented neopterin in these patients may indicate autonomous compartmentalized HIV infection within CNS macrophages [46], whereas infection in the non-ADC patients may be largely transitory, non-compartmentalized and supported within lymphocytes with less robust stimulation of macrophages. Of course, these associations need to be more directly established, but they provide an attractive bridge between these observations on neopterin and virological studies showing that virus detected in CSF likely has at least two origins [47,48].

## CSF Neopterin in Clinical Management of HIV Infection

Given the changes in CSF neopterin and its relation to the critical process of immunoactivation within the CNS, one can ask whether there might be a role for measurement of this CSF biomarker in clinical practice, including diagnosis, prognosis and treatment evaluation related to CNS injury.

### Diagnosis

When HIV-infected patients present with neurological abnormalities, the character of symptoms and signs leads to appropriate evaluations for opportunistic infections, malignancies, vascular diseases and other afflictions using neuroimaging and other modalities. Absence of focal clinical or neuroimaging toxoplasmosis/CNS lymphoma findings, and negative CSF analysis for CMV, other herpes virus, JCV, EBV and cryptococcus supports the ADC/HIVE diagnosis.

**Figure 6:**
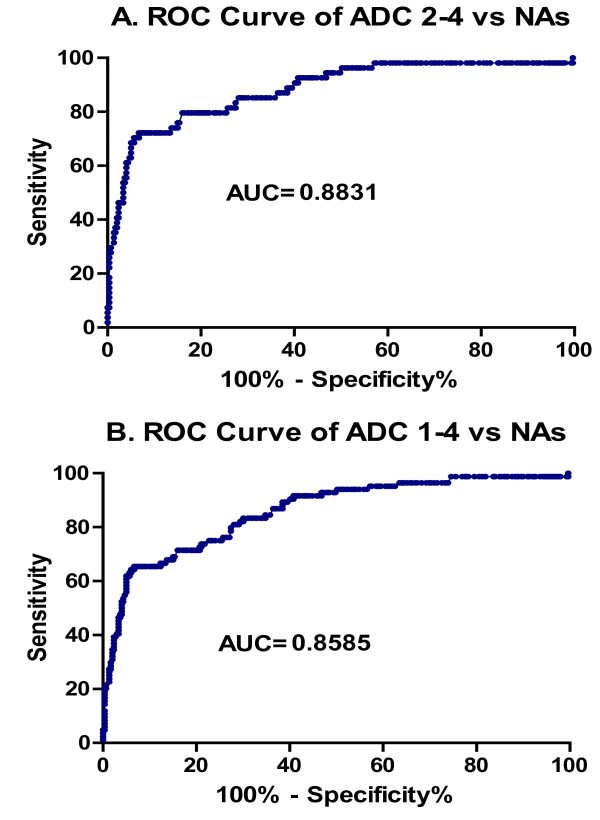
**ROC curves for two comparisons**. A. ADC 2-4 was compared to the four groups of untreated neuroasymptomatics. B ADC 1-4 (ADC 1 group and 2-4 group combined) was compared to the four groups of NAs. AUC, area under the ROC concentration curve where 1 is high and 0.5 not different from random.

To assess the value of CSF neopterin in this setting, we used the cross-sectional study results shown in Figure 2. For this analysis we excluded CNS opportunistic infections, though one should caution that, especially CMV-encephalitis, cryptoccal meningitis and CNS lymphoma, may also elevate CSF neopterin to levels seen in patients with ADC. Using this cross-sectional study data, we constructed a series of receiver-operator characteristic (ROC) curves to estimate the sensitivity and specificity of CSF neopterin in the diagnosis of ADC. Figure 6 shows two of these in which ADC 2-4 (A) and ADC 1-4 (B) were compared to the four groups of untreated neuroasymptomatic subjects. From this analysis, if one uses a cutoff of CSF neopterin ≥ 30 nmol/L, the sensitivity for a diagnosis of ADC 2-4 is 80% and the specificity 81% (likelihood ratio = 4.2). For ADC 1-4 the sensitivity drops to 71%, while the specificity remains at 81% (likelihood ratio = 3.8). A higher cut off of 40 nmol/L yields sensitivity for ADC 2-4 of 72%, specificity of 93% and likelihood ration of 9.8, while for ADC 1-4 the sensitivity of 66%, specificity again 93% and the likelihood ratio is 8.9.

Thus, measuring the CSF neopterin has diagnostic value in ADC, though not to a degree to provide sufficiently certain diagnosis on its own. The reasons for this uncertainty relate principally to the overlap of the neuroasymptomatic subjects into the range of the ADC subjects, particularly the ADC 1 group. There are several explanations for this beyond a true biological overlap in CSF neopterin. These include imprecision of clinical diagnosis in classifying our subjects. Thus, some of the neuroasymptomatics may indeed have had incipient or unrecognized brain injury. Case D provides an example where CSF neopterin elevation indeed predicted clinical presentation. On the other hand, some patients diagnosed as ADC might have suffered other conditions. At the present time there is no objective 'gold standard' for this diagnosis.

### Prognosis

Is it possible to use CSF neopterin concentrations as a prognostic marker? In a prospectively studied cohort of 35 neurologically asymptomatic HIV-infected patients, CSF neopterin above 20 nmol/l had almost 7 times the risk of developing ADC, but the risk did not increase further when CSF neopterin was above 40 nmol/L [16]. These patients were neurologically asymptomatic at inclusion but had advanced HIV infection as measured by CD4+ cell count (<200 cells/μl) and the median follow-up time was 21 months. In a longitudinal retrospective study with a longer follow up time CSF neopterin concentration did not predict dementia development in 8 patients compared with matched controls, although these patients had higher CD4 cell count [49]. In the same study, however, the neurofilament light chain protein (NFL), a CSF biomarker of axonal injury, predicted dementia development [49]. Further studies comparing markers and using marker combinations may help to clarify this issue.

### Treatment effect

The goal for antiretroviral treatment is to eliminate mortality and morbidity related to organ dysfunction, including the CNS, related directly or indirectly to HIV infection. We generally measure this efficacy using the surrogate, plasma HIV RNA level. However, there is growing concern that CNS morbidity can continue despite treatment that suppresses plasma, and even CSF, viremia, at least as measured by conventional clinical assays [50]. Can CSF neopterin provide a more refined measure of successful amelioration of CNS infection and, more particularly, ongoing CNS injury?

Combination ART has a profound effect on CSF viral load and neopterin levels as shown above and reported previously [51]. Hence, looking at the positive side, CSF neopterin is reduced by therapy to levels below those of asymptomatic infection and well below those characteristic of ADC. However, looking at the 'half-empty' side, these levels often remain above normal. Does this indicate ongoing infection and should further efforts be made in the individual patient to assure that they indeed return to normal? Some antiretroviral drugs appear to be more effective in reducing CSF viral load and possibly CNS immunoactivation, and it has been suggested that CNS drug penetration may be an important aspect of treatment in general [41]. Our results failed to show a relationship between the CPE scores that take into account CNS drug penetration and CSF neopterin levels. While our study was not designed to test this issue and there were few subjects in the lower score range, these results may suggest that other drug properties are important in determining the effect on CNS immunoactivation in this setting.

Persistent low-level neopterin production might per se be associated with chronic CNS damage, as expression of intrathecal immune activation and also because of its probable neurotoxic effect. Given the long lifespan of HIV-infected persons, these effects might, in the long-term, combine with CNS insults typical of the older age and contribute to functional neurological impairment.

## Conclusions

Combination antiretroviral drug treatment has had a dramatic effect on morbidity and mortality of HIV infection, including those involving the CNS and the previously most common of these, ADC/HIVE. The study of CSF neopterin has contributed to our understanding of CNS HIV infection and its consequences. As attention now turns to the potential CNS effects of lower grade immunoactivation before or in the presence of treatment, continued study of CSF neopterin may turn out to be helpful in this next phase of understanding pathogenesis and designing and evaluating therapeutics. We do not understand the consequences of chronic immune activation in these settings and whether interventions can be tailored to reduce any deleterious effects. Hence, further studies are needed to evaluate this as well as whether particular antiretroviral drug combinations may more effectively minimize such immune activation. Although nonspecific, we think that neopterin concentration is a useful biomarker in monitoring this CNS immune activation and its potential consequences, and in evaluating the effects of different antiviral and even adjuvant strategies that have proved so difficult to assess using neurological symptoms or signs, neurocognitive performance or CSF viral loads. CSF neopterin may prove to be a valuable surrogate to address these important issues.

## Declaration of interests

The authors declare that they have no competing interests.

## Authors' contributions

LH, PC, RWP, MG, BJB, SS, and AB collected CSF samples and made the subject evaluations. DF and RWP were responsible for the biochemical analyses. The study was planned and interpreted and the data were reviewed and revised by all the authors. LH and RWP prepared the manuscript. All authors read and approved the final manuscript.
